# A Time-Varying Information Measure for Tracking Dynamics of Neural Codes in a Neural Ensemble

**DOI:** 10.3390/e22080880

**Published:** 2020-08-11

**Authors:** Mohammad R. Rezaei, Milos R. Popovic, Milad Lankarany

**Affiliations:** 1 Division of Clinical and Computational Neuroscience, Krembil Research Institute, University Health Network, Toronto, ON M5T 0S8, Canada; m.reza.rezaei72@gmail.com; 2 KITE Research Institute, Toronto Rehabilitation Institute-University Health Network, Toronto, ON M5G 2A2, Canada; milos.popovic@uhn.ca; 3 Institute of Biomedical Engineering, University of Toronto, Toronto, ON M5S 3G9, Canada

**Keywords:** information-theoretic measure, rate code, temporal code, multiplexed code, asynchronous and synchronous spikes

## Abstract

The amount of information that differentially correlated spikes in a neural ensemble carry is not the same; the information of different types of spikes is associated with different features of the stimulus. By calculating a neural ensemble’s information in response to a mixed stimulus comprising slow and fast signals, we show that the entropy of synchronous and asynchronous spikes are different, and their probability distributions are distinctively separable. We further show that these spikes carry a different amount of information. We propose a time-varying entropy (TVE) measure to track the dynamics of a neural code in an ensemble of neurons at each time bin. By applying the TVE to a multiplexed code, we show that synchronous and asynchronous spikes carry information in different time scales. Finally, a decoder based on the Kalman filtering approach is developed to reconstruct the stimulus from the spikes. We demonstrate that slow and fast features of the stimulus can be entirely reconstructed when this decoder is applied to asynchronous and synchronous spikes, respectively. The significance of this work is that the TVE can identify different types of information (for example, corresponding to synchronous and asynchronous spikes) that might simultaneously exist in a neural code.

## 1. Introduction

The collective responses of primary sensory neurons constitute fully or partly mixed inputs to cortical neurons; thus, multiple features of the stimulus are to be reliably coded by cortical neurons. The brain uses different coding strategies to represent information underlying those features. Information can be encoded by either the rate of spikes in a relatively long time window—rate code—or by their precise timing—temporal code [1,2,3,4,5,6,7,8,9]. In temporal coding, information is mostly carried by groups of neurons that fire nearly simultaneously [9,10] (see [11] for other forms of temporal coding) whereas, in the rate coding, the precise timing of spikes is compromised and information across neurons is mostly carried by the rate of asynchronous spikes [12,13,14,15].

It has been suggested that the presence of both coding strategies, whose feasibility was demonstrated in different neural systems (e.g., [16,17]), can be considered as a unique way to convey multiple features of the stimulus, i.e., multiplexed coding. In fact, in addition to the rate code, which is largely observed in different neural systems, inter-neuronal correlations within many areas of the brain have a significant functional role in the neural code [18,19,20]. Temporal correlations between neurons can contribute to coding additional information, which are not represented by the isolated spike trains. However, it remained unknown to what extent these coding strategies cooperatively contribute to the representation of a mixed stimulus. It is challenging to uncover the distinct roles of differentially correlated spikes—i.e., asynchronous spikes (rate code) and synchronous spikes (temporal code)—in a multiplexed code. To address this challenge, it is crucial to measure the information underlying different types of spikes [16]. Various information-theoretic techniques have been exploited to measure information carried by differentially correlated spikes [15,21]. These methods can be classified into two categories, namely, direct and indirect approaches [19]. In the indirect approach, information is calculated based on the relationship between the stimulus and neural responses. In contrast, in the direct approach, information is obtained based on the statistics of the neural responses, regardless of any assumptions about the stimulus [19]. For example, in the indirect approach, mutual information (MI) between the stimulus and spikes [22,23,24] is calculated based on their joint probability distribution [23,25,26]. Thus, the computational complexity is expensive. Although some methods, like non-parametric kernel estimation [27] and Gaussian approximation [28], are used to reduce the computational complexity of calculating the joint distribution, the indirect approaches are not sufficiently accurate when applied to multi-dimensional neural activity, i.e., spikes in a neural ensemble [29]. In contrast, information can be measured with less complexity in the direct approaches.

It is worth mentioning that information, in almost all the existing methods, is calculated by the length of the stimulus interval. Nevertheless, the information of a mixed stimulus might be represented by spikes at different time scales. The rate-modulated asynchronous spikes and precisely correlated synchronous spikes occur in different timescales. Thereby, to calculate information in a multiplexed code, it is important to calculate information over time (at each time bin) and in different time scales. In this paper, we propose a time-varying entropy (TVE) measure that calculates the entropy of a neural ensemble in response to a mixed stimulus consisting of slow and fast signals. The simultaneous representation of these signals through synchronous and asynchronous spikes was recently demonstrated [16]. Inspired by [19], we consider spikes as code words with different lengths and time resolutions, and calculate the entropy across homogeneous neurons in a neural ensemble. In this way, we estimate the entropy of spikes at each time bin and show how it varies in different time resolutions, which correspond to different features of the stimulus. Furthermore, by computing the probability distributions and entropies of asynchronous and synchronous spikes in a neural ensemble, we show that these spikes carry different information. The TVEs underlying synchronous and asynchronous spikes have their maximum values when the code words are selected with specific time resolutions. In addition, we demonstrate that the TVEs of synchronous and asynchronous spikes are highly correlated with the fast and slow signals, respectively. Finally, we use a Kalman decoder to reconstruct these features of the stimulus using asynchronous and synchronous spikes. Our results indicate that information underlying synchronous and asynchronous spikes is different and associated with distinct features of the stimulus.

## 2. Computational Framework

### 2.1. Responses of a Homogeneous Neural Ensemble to a Mixed Stimulus

According to the feasibility of neural systems to multiplexed coding [16,30], we simulated the activity of a homogeneous neural ensemble in response to a mixed stimulus to explore how much information can be encoded by different patterns of spikes. Each neuron received a mixed signal $\left(I_{mixed}\right),$ which consists of a fast signal $\left(I_{fast}\right)$ and a slow signal $\left(I_{slow}\right)$ [16]. $I_{fast}$ stands for the timing of fast events or abrupt changes in the stimulus and was generated by convolving a randomly (Poisson) distributed Dirac-delta function with a synaptic waveform (normalized to the peak amplitude), $\tau_{rise}=0.5\;\mathrm{ms}$, and $\tau_{fall}=3\;\mathrm{ms}$. Fast events occurred at a rate of ∼1 Hz and were scaled by $a_{fast}=85\;\mathrm{pA}$.

$I_{slow}$ was generated by an OU process as follows

(1) $$\frac{dI_{slow}}{dt}=-\frac{I_{slow}\left(t\right)-\mu }{\tau }+\sigma \frac{\sqrt{2}}{\tau }\xi \left(t\right),\;\;\xi \sim Ν\;\left(0,1\right)$$

where ξ is a random number drawn from a Gaussian distribution, τ=100 ms is the time constant of the slow signal that produces a slow-varying random walk with an average of µ=15 pA and a standard deviation of σ=60 pA. The mixed signal $\left(I_{mixed}\right)$ was obtained by adding $I_{fast}$ and $I_{slow}$, which are generated independently.

An independent noise (equivalent to the background synaptic activity) was added to each neuron, thus each neuron receives a mixed signal plus noise. Similar to [31], the noise ($I_{noise}$) was generated by an OU process of τ=5 ms, µ=0 pA, and σ=10 pA.

**Figure 1:**
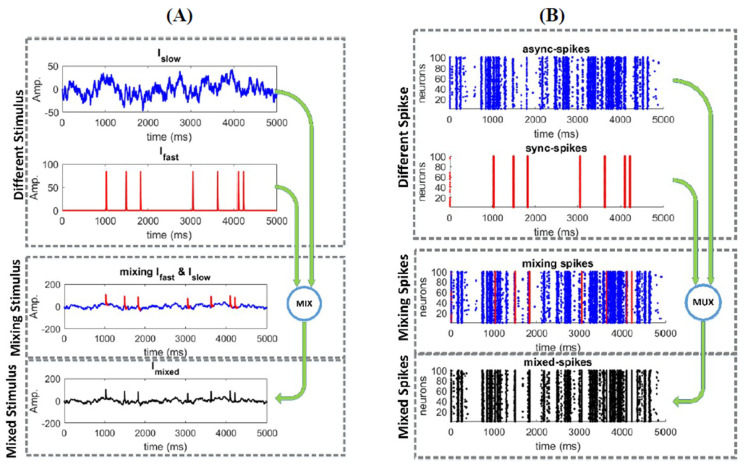
The simulation data consist of the mixed stimulus and spiking activity of the neural ensemble. (**A**) The mixed stimulus $I_{mixed}$ consists of $I_{fast}$ and $I_{slow}$ (see Section 2) (**B**) Different patterns of spikes resulted from the neural ensemble (including 100 neurons) as response to a mixed stimulus.

The neural ensemble consists of 100 neurons, each of them was modeled by Morris–Lecar equations [32,33]. The equations of a single model neuron receiving a mixed-signal plus noise can be written as follows

(2) $$C\frac{dV}{dt}=I_{mixed}\left(t\right)+I_{noise}\left(t\right)-{\bar{g}}_{Na}m_{\infty }\left(V\right)\left(V-E_{Na}\right)-{\bar{g}}_{K}w\left(V-E_{K}\right)-g_{L}\left(V-E_{L}\right)\;-{\bar{g}}_{AHP}z\left(V-E_{K}\right)-g_{exc}\left(V-E_{exc}\right)-g_{inh}\left(V-E_{inh}\right)$$

where,

(3) $$\frac{dw}{dt}=\phi \frac{w\left(V\right)-w}{\tau_{W\left(V\right)}}$$

(4) $$\frac{dz}{dt}=\frac{\frac{1}{1+e^{(\beta_{z}-V)/\gamma }\;}-z}{\tau_{z}}$$

(5) $$m_{\infty }\left(V\right)=0.5\left[1+\tanh\left(\frac{V-\beta_{m}}{\gamma_{m}}\right)\right]$$

(6) $$w_{\infty }\left(V\right)=0.5\left[1+\tanh\left(\frac{V-\beta_{w}}{\gamma_{w}}\right)\right]$$

(7) $$\tau_{w}\left(V\right)=\frac{1}{\cosh\left(\frac{V-\beta_{w}}{2\beta_{w}}\right)}$$

where $\;\left\{{\bar{g}}_{Na}=20,\;{\bar{g}}_{k}=20,\;{\bar{g}}_{L}=20,\;{\bar{g}}_{AHP}=25,\;g_{exc}=1.2,\;g_{inh}=1.9\right\}\frac{\mathrm{ms}}{{\mathrm{cm}}^{2}}$, $\{E_{Na}=50,E_{K}=-100,\;E_{L}=-70,\;E_{exc}=0,E_{inh}=-70\}\mathrm{mV},\;\beta_{m}=-1.2,\;\gamma_{m}=18,\;\beta_{w}=-19,\;\gamma_{w}=10,\;\beta_{z}=0,\;\gamma_{z}=2$, $\tau_{a}=20\;\mathrm{ms},\;\phi =0.15,\;and\;C=2\frac{\mu F}{{\mathrm{cm}}^{2}}.$ These parameters were set to ensure a neuron operates in a hybrid mode [34], i.e., an operating mode between integration and coincidence detection [35]. The inclusion of background excitatory and inhibitory synaptic conductance in (2) reproduced a “balanced” high-conductance state [36]. The surface area of the neuron was set to $200{\;µm}^{2},$ so that $I_{mixed}$ is reported in pA, rather than as a density. Figure 1 shows different steps towards constructing the mixed signal and stimulating a neural ensemble. Figure 1A shows how the mixed signal was made by $I_{slow}$ and $I_{fast}$ signals. The spiking activity of the neural ensemble is shown in Figure 1B. Similar to [16], synchronous spikes, sync-spikes, and asynchronous spikes, async-spikes, were distinguished based on a synchrony threshold. Therefore, the dataset consists of the mixed-stimulus and the spiking activities of the neural ensemble where different elements of the mixed stimulus ($I_{fast}$, $I_{slow}$) and their related neural activities were shown in different colors.

### 2.2. Probability Density Estimation

We used a histogram-based method [37] to calculate the probability distributions of spiking activities or word patterns. We considered 100 bins (as 100 is the total number of neurons used in the simulation study) for the construction of the histograms of different types of spikes. For each word pattern, we considered $2^{L}\;$ bins, where L is the length of the word pattern, to construct histograms that include all possibilities. Finally, the histograms are normalized to reach a probability density function.

## 3. Results

### 3.1. Information Underlying Synchronous and Asynchronous Spikes Are Distinctively Separable

To address whether synchronous and asynchronous spikes convey different information, we test if these spikes are distinctively separable [27]. We use mutual information (MI) to measure the similarities between the probability distributions of these spikes [27,28], i.e., I(A;S), where S and A are random variables drawn from the distributions of synchronous and asynchronous spikes, respectively. The MI can be written as follows [38]

(8) $$I\left(A;\;S\right)=\underset{a\in A}{\sum }\underset{s\in S}{\sum }p_{\left(A,S\right)}\left(s,a\right)\log\left(\;\frac{p_{\left(A,S\right)}\left(s,a\right)}{P_{A}\left(a\right)P_{S}\left(s\right)}\right)$$

where $P_{S}\left(s\right)$ and $P_{A}\left(a\right)$ are the distributions of synchronous and asynchronous spikes, respectively, and $p_{S,A}\left(s,a\right)$ is the joint probability distribution of synchronous and asynchronous spikes. We utilized the histogram-based method suggested in [37] with 100 bins to calculate the probability distributions of each type of spikes. The probability at each bin in the histogram is equal to the number of counts divided by the total number of counts in the histogram. *I* (≥0) is equal to zero if the distributions of sync-spikes ($P_{S}\left(s\right))$ and async-spikes ($P_{A}\left(a\right)$) are independent. To precisely demonstrate the difference between probability distributions of synchronous and asynchronous spikes, we used a non-parametric method to estimate these distributions [39]. This method estimates the probability density function by using normal kernel smoothing function and a bandwidth as follows

(9) $${\hat{f}}_{h}\left(x\right)=\frac{1}{Nh}\overset{N}{\underset{i=1}{\sum }}K(\frac{x-x_{i}}{h})\;;-\infty <x<\infty$$

where ${\hat{f}}_{h}\left(x\right)\;$ is the approximated histogram, *N* is the sample size (2 × 10^5^ samples of data in the simulation), *K*(.) is the kernel function, and *h* is the bandwidth, which is fixed on 0.4 based on the smoothness of the data.

**Figure 2:**
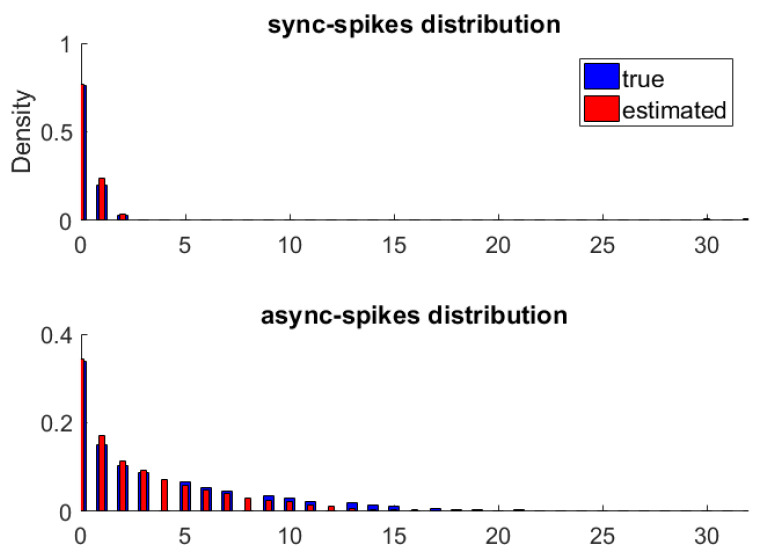
The probability distributions of synchronous (top) and asynchronous (bottom) spikes. For each type of spike, the true distribution was obtained by the histogram method and is shown by blue bars (thick bars). We used a non-parametric method to approximate distributions of synchronous and asynchronous spikes, which are shown by red bars (thin bars).

Figure 2 shows the original and approximated probability distributions of synchronous and asynchronous spikes. The MI between synchronous and asynchronous spikes is nearly zero (I=0.003 and  0.015 for histogram-based and non-parametric methods, respectively), suggesting that the statistical dependencies between their probability distributions are negligible.

We also used a statistical hypothesis test to quantify statistical differences between synchronous and asynchronous spikes. A two-sample version of the Kolmogorov test [40,41] was used to detect a wide range of differences between the two distributions. In this way, one can compare the distribution functions of the parent populations of the two samples drawn from the distributions of synchronous and asynchronous spikes. The null hypothesis is that these samples are drawn from an identical distribution function. The statistical test (repeated 1000 times) rejected the null hypothesis at the default significance level of 5% for both histogram-based and non-parametric methods. Our analysis shows that synchronous and asynchronous spikes have different and separable statistical characteristics, which might lead to encoding different types of information.

### 3.2. Different Types of Spikes in a Multiplexed Code Carry Different Amounts of Information

**Figure 3:**
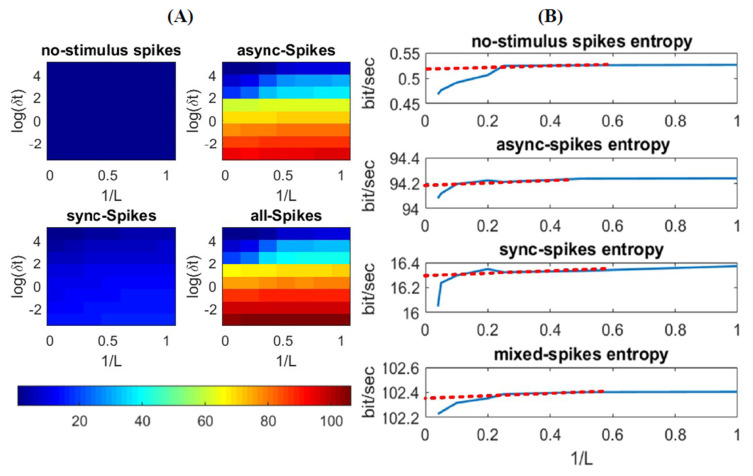
Entropy of different types of spikes. (**A**) Entropy of different patterns of spikes as a function of time-bin (δt) and word-length (L) (**B**) Estimated entropy rate of spikes, for no-stimulus, slow, fast, and mixed stimuli, is plotted against the reciprocal of word length, 1/*L*. The dashed line and its intersection with y axis represent the value of entropy for L→∞, i.e., the minimum value of the entropy.

To quantify the amount of information each type of spike carries, we measure the entropy of synchronous and asynchronous spikes. The entropy determines the variability underlying the probability distributions of spikes [27] and indicates the upper bound of information of spikes. Similar to [42], we considered neural responses as binned spike trains and calculated the entropy of short strings of bins, or words, for each individual neuron. This estimation of entropy depends on two parameters, namely, temporal resolution (δt) and temporal structure or word length (L). The entropy, H(L,δt), is defined as follows [19]

(10) $$H\left(L,\delta t\right)=-\frac{1}{L\delta t}\;\underset{w\in W\left(L,\delta t\right)}{\sum }p\left(w\right)\log_{2}p\left(w\right)$$

where w is a specific word with length L; W(L,δt) is the set of all possible words comprising L bins, and p(w) is the probability of observing a word w in the neural response. The advantage of this method, in comparison to other information measures like the mutual information between stimulus and spikes [27], is that this method is a direct way to estimate information of the spikes, with no need to access the stimulus. After distinguishing synchronous and asynchronous spikes in a neural ensemble (see Section 2), we calculated H(L,δt) of each individual neuron for different word lengths (L) and time-bin resolutions (δt) to assess the effect of these variables in extracting information underlying each type of spikes. Figure 3 shows the average of the entropy of individual neurons. The entropy decreases for a low time resolution (i.e., high δt) due to a higher temporal correlation between spikes in a large time bin compared to that in short time bins (see the gradual color contrast vertically in Figure 3A). In addition, the entropy, H, decreases by increasing  L due to the integration of dependencies among the time bins, i.e., the longer the word length is, the less uncertain the code word is (see Figure 3B). Given enough data samples for estimating p(w), for an infinite length of L, the entropy can be calculated when δt→0 and L→ ∞ [19]. However, due to the finite length of data, which leads to a finite length of L, we extrapolated the entropy for an optimum δt (i.e., 0.05 ms) and L→ ∞, and we found a steady-state rate of entropy for different types of spikes (red lines in Figure 3B). As shown in this figure, synchronous and asynchronous spikes convey different rates of entropy (the steady-state rate of entropies for synchronous and asynchronous spikes are about 16.2 and 94.2 bit/s, respectively). In addition, the entropy of all spikes (calculated as the average of the entropy of individual neurons) is about 102 bit/s. The interpretation of an entropy measure of 102 bit/s is that the spiking activity of the neural ensemble can carry as much information as would be required to perfectly discriminate $2^{102}$ different 1-s-long samples of the stimulus [19]. In the next section, we see if such difference in information measure between synchronous and asynchronous spikes is associated with different features of the stimulus.

### 3.3. Time-Varying Entropy (TVE) Measure

To determine how information of spikes are related to different features of the stimulus, we propose an entropy measure, namely, time-varying entropy (TVE), to calculate the entropy of spikes in a neural ensemble at each time bin. TVE is defined as follows

(11) $$H\left(L,\delta t,\;k\right)=-\frac{1}{L\delta t}\underset{w\in W_{k}\left(L,\delta t\right)}{\sum }p\left(w\right)\log\left(w\right)$$

where k  is the index of the time bins and p(w) is the probability of a specific word with length L at time k across the neurons (trials). $W_{k}\left(L,\delta t\right)$ is a set of all possible words with length L and time resolution δt at time-step k across trials. The TVE, in (11), is calculated across neurons and introduces a time-varying entropy measure for an ensemble of neurons. The main difference between the entropy in (10) and that in (11) is that in the former the expected value of (logarithm of) code words is obtained over the length of stimulus, whereas in the latter, it is calculated across neurons, thus providing an entropy measure over time. In words, the entropy in (11) is considered as an information-theoretic measure to calculate information underlying spikes.

**Figure 4:**
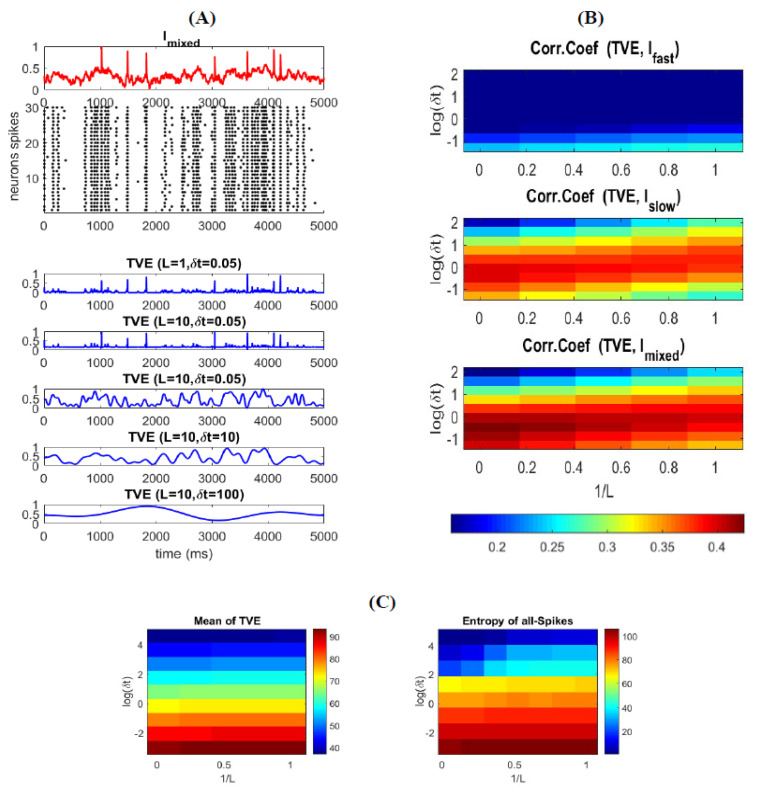
Time-varying entropy (TVE) measure for different type of spikes. (**A**) TVE for different type of spikes as function of word-length and time-bins resolution. Different parameter sets for TVE enables extracting different types of information. For example, by setting L=10, δt=0.05 ms TVE extracts information underlying synchronous spikes. As well, by setting L=10, δt=10 ms  TVE extracts information related to asynchronous spikes (**B**) TVE measure correlation coefficient with $I_{fast}$, $I_{slow}$, and $I_{mixed}$. The correlation of TVE with each stimulus is aligned with the figures in panel (**A**). For L=10, δt=0.05 ms  TVE is highly correlated with $I_{fast}$, which drives synchronous spikes. For L=10, δt=10 ms  TVE is highly correlated with $I_{slow}$, which provokes asynchronous spikes. Thus, TVE measure can extract information about the stimulus directly from the spikes (**C**) Mean of Integration of TVE measure over time (left) and entropy of all-spikes calculated in Equation (9) (right).

To explore the relationship between (all) spikes and the stimulus features, we calculate the correlation between the TVE and stimulus for different combinations of word-lengths (L) and time resolutions (δt). To better visualize how the entropy changes over time, we plotted a few examples of TVE for different L and δt in Figure 4A. Figure 4B shows the relationship between TVE and different features of the stimulus as well as the mixed stimulus as a function of L and δt. As can be seen in this figure, TVE is highly correlated with the $I_{fast}$ for small time bins, implying that the neural code of a neural ensemble utilizes spikes with a very high temporal resolution to represent fast (abrupt) changes in the stimulus (see also Figure 4A for L=1&10 and δt=0.05 ms). For relatively high temporal resolution, TVE increases slightly for shorter L (Figure 4B (top)), confirming that precise timing of spikes is sufficient to represent fast features of the stimulus. Although TVE is calculated for all spikes, one can interpret that spatially correlated spikes in a short time interval in a neural ensemble are considered as synchronous; thus, the code words of L = 1 provide better representation for synchronous spikes (i.e., code words are temporally independent).

Figure 4B (middle) shows that the TVE is highly correlated with the $I_{slow}$ for medium time bins, indicating that the neural code of a neural ensemble uses spikes in a relatively low temporal resolution to encode the amplitude of smooth changes (low frequency) in the stimulus (see also Figure 4A for L=10 and δt=5&10 ms). For relatively medium temporal resolution, TVE increases slightly for longer L, suggesting that an appropriate range of temporal correlation within the code words enhances the information representation of the slow features of the stimulus. Figure 4B (bottom) shows the correlation of TVE and the mixed stimulus ($I_{mixed})$.

Unlike (10), where the entropy is calculated for each individual neuron (over the total stimulation time), the TVE computes the entropy of a neural ensemble over time (at each time bin). One can expect that the average of the TVE over time is equivalent to the average of entropy of individual neurons (as calculated in (10)). Figure 4C shows that the average of the TVE over time is similar to that of individual neurons. As mentioned above, the entropy of all spikes based on (10), and in agreement with [19], is 102 bit/s. As well, the integral of the TVE over time (for the same L and δt) is equal to 92.58 bit/s. It is to be noted that the time-varying entropy in (11) is calculated based on the probability distribution of spikes (for each time-bin) across a limited number of trials (neurons). Therefore, one can expect that the entropy calculated by [19] provides an upper bound of time-varying entropy that an ensemble of neurons with a limited number of neurons can carry.

**Figure 5:**
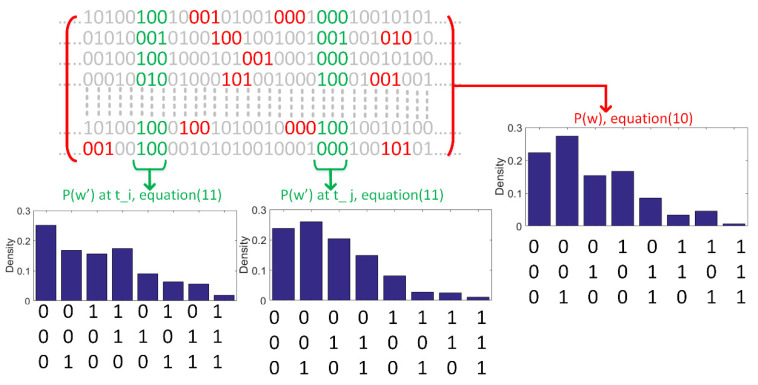
Illustration of calculation of the entropy in (10) and the TVE in (11). The binary sequence of each row indicates the response of each neuron in a neural ensemble. Probability distribution of code words, *p*(*w*), over the whole length of data can be calculated based on (10). Two probability distributions underlying two time bins, *t*_i_ and *t*_j_, are calculated across neurons (see (11)). The length of code words is equal to 3 and spikes are binned at a resolution (δt), equal to the sampling time of the simulation. Several code words are highlighted by red and green.

To better clarify the difference between the entropy in (10) and the TVE in (11), we illustrate in Figure 5 how the entropy is calculated across trials (neurons) for any given time. For specific L and δt, the probability distribution of code words, *p*(*w*), can be calculated over the whole simulation time (see (10)). To calculate the TVE, the probability distribution of code words, for any given time, can be calculated across neurons. Figure 5 shows two probability distributions of code words in different time bins, namely, *p*(*w*’) at *t*_i_ and *t*_j_.

**Figure 6:**
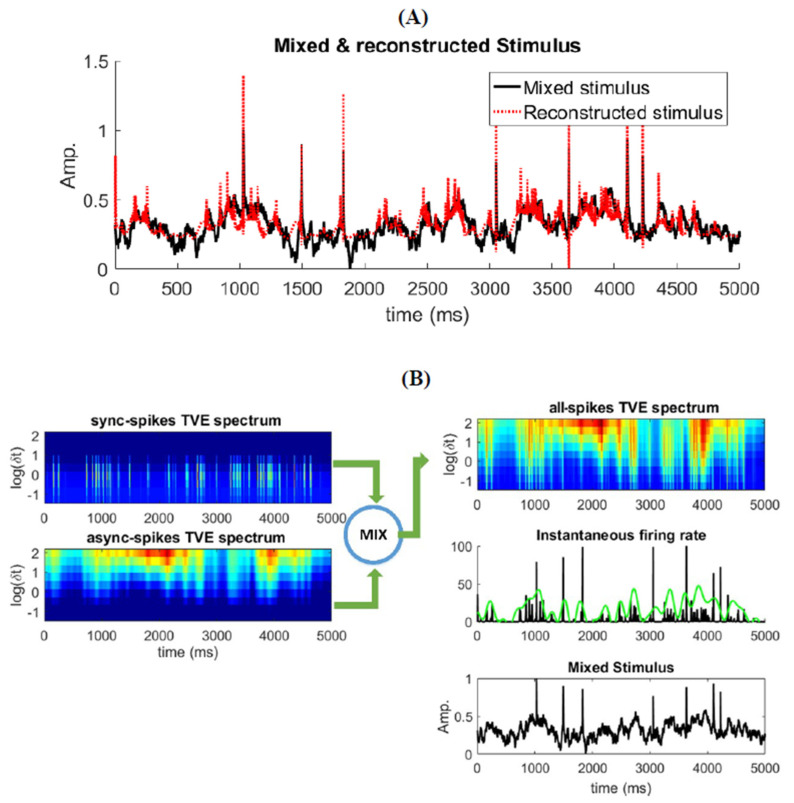
Different elements of the mixed stimulus ($I_{fast}$, $I_{slow}$) and their relationship with different type of spikes. (**A**) Reconstruction of the mixed stimulus by TVE measure. (**B**) TVE measure spectrums for different patterns of spikes (synchronous, asynchronous, and all) given different δt with fixed L=10 through time. Instantaneous firing rate of the neural ensemble is calculated with two different kernel width; green and black graphs are related to kernel width = 100 ms and kernel width = 5 ms, respectively.

Furthermore, to calculate the optimum values of L and δt, which lead to extract maximum information of the mixed stimulus, we build a linear decoder model to reconstruct the stimulus from different combinations of TVE measures. We used a linear regression model with root mean squared error (RMSE) cost function [43] to calculate the linear coefficients and parameter setting of the TVEs. The linear decoder model and the cost functions are written as

(12) $$\begin{array}{c} \hat{y}=w_{s}*{\mathrm{TVE}}_{s}+w_{a}*{\mathrm{TVE}}_{a}+b;\;\;\;{\mathrm{TVE}}_{s}=\mathrm{TVE}\left(L_{s},\delta t_{s}\right),\;\;\\ {\mathrm{TVE}}_{a}=\mathrm{TVE}\left(L_{a},\delta t_{a}\right) \end{array}$$

(13) $$\left\{w_{s},w_{a}\right\}=argmin_{\left\{w_{s},w_{a\;},\;b,\;\;L_{s},\;\;\delta t_{s},\;\;L_{a},\;\;\delta t_{a}\right\}}\;\sqrt{\frac{1}{N}\overset{N}{\underset{k=1}{\sum }}{\left(y_{k}-{\hat{y}}_{k}\right)}^{2}}$$

where ${\mathrm{TVE}}_{s}$ and ${\mathrm{TVE}}_{a}$ are the TVE measures for spikes with $\left\{L_{s},\delta t_{s}\right\}$ parameters set and those with the $\left\{L_{a},\delta t_{a}\right\}$ parameters set, respectively. N is the total number of samples, $y_{k}$ and ${\hat{y}}_{k}$ are the mixed and estimated stimulus at time index k, and $\left\{w_{s},w_{a},\;b\right\}$ are the regression parameters for ${\mathrm{TVE}}_{s}$ and ${\mathrm{TVE}}_{a}$, respectively. We optimize the linear decoder model for different parameter settings for ${\mathrm{TVE}}_{s}$ and ${\mathrm{TVE}}_{a}$, and select the optimum decoder based on its RMSE performance. Figure 6A shows the true and reconstructed mixed stimulus by (12). The optimum values of *L* and δt (not shown here), for ${\mathrm{TVE}}_{s}$ and ${\mathrm{TVE}}_{a}$, to reach the best decoding performance are the same as the parameters presented in Figure 4B, for which the highest correlation between TVE (all spikes) and fast and slows signals was obtained. These results justify that TVE with specific (optimum) ranges of δt and L  corresponds to different types of information underlying distinct features of the stimulus.

We can represent this relationship clearly by visualizing the TVE measure spectrum for different δt and L through time (note that TVE is less sensitive to the changes in *L* compared to that in δ; see (Figure 4A). The TVE can identify which information is carried by spikes and reconstructs its associated stimulus features. Figure 6B shows the TVE calculated for synchronous, asynchronous spikes, and all spikes for different δt and a fixed L (=10). One can clearly observe that TVE calculated by asynchronous spikes represents information of slowly time-varying changes in the stimulus for medium to high δt. In contrast, the TVE obtained by synchronous spikes represents information underlying abrupt changes in the stimulus for small δt. Therefore, the TVE calculated by all spikes and for different δt creates a heat map of information (i.e., the TVE spectrum) underlying different features of the stimulus. It is worth mentioning that by integrating the TVE over time (similar to Figure 4C) for synchronous and asynchronous spikes, one can measure how much information is carried by each type of spike.

**Figure 7:**
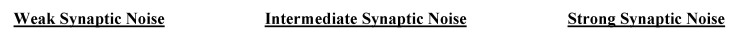

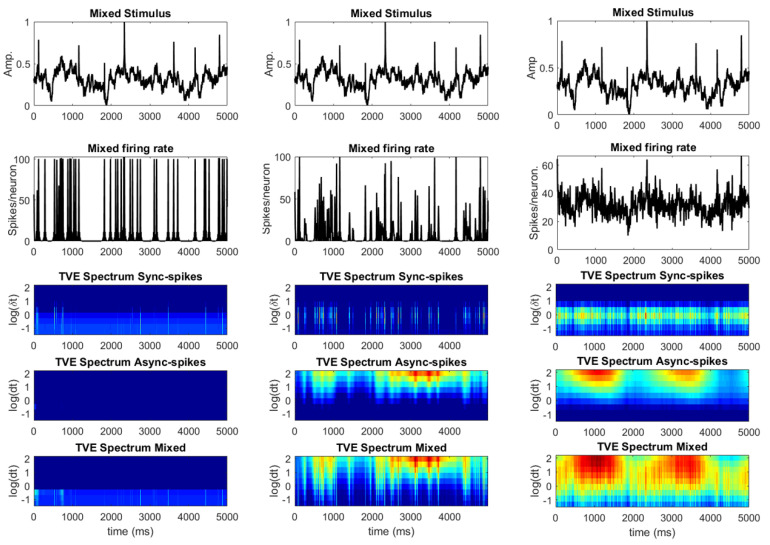
Firing rate and TVE spectrum of spikes for a neural ensemble receiving weak (left, σ=0.5 pA), intermediate (middle, σ=10 pA), and strong (right, σ=50 pA) synaptic noises. The TVE spectrum of different types of spikes is obtained in a similar way, as explained in Figure 6.

Although the TVE spectrum in Figure 6 reveals that synchronous and asynchronous spikes are decodable in different time-resolution scales, the extent to which these spikes can represent the stimulus features relies on multiple factors like the level of background synaptic noise, network size, intrinsic parameters of single neurons, etc. For example, a recent study [44] investigated the necessary conditions underlying reliable representation (and propagation) of time-varying firing rates in feed-forward neural networks with homogeneous neurons. It has been shown that a proper and biologically realistic level of background synaptic noise is substantial to preserve information of a common stimulus. To explore how the level of background synaptic noise alters the co-existence of decodable synchronous and asynchronous spikes (i.e., multiplexing), we consider two extreme cases in which a neural ensemble receives weak and strong synaptic noise. Figure 7 (two top rows) shows the stimulus and the firing rate of a neural ensemble receiving weak (σ=0.5 pA), intermediate (σ=10 pA), and strong (σ=50 pA) synaptic noise. The neural response tends towards synchronous states for a low level of background synaptic noise (Figure 7 (left, second row)). In contrast, the neural response converges to the average firing rate (with some fluctuations) for a high level of synaptic noise (Figure 7 (right, second row)). Therefore, multiplexing—in the sense of decodable synchronous and asynchronous spikes—fails in these extreme cases (see [44] for more details).

For each level of background synaptic noise, the TVE spectrum (similar to Figure 6) is calculated for synchronous, asynchronous, and all spikes. That of synchronous spikes fully represents the TVE spectrum of a neural ensemble receiving weak synaptic noise (see Figure 7 (left, last three rows)). In contrast, the TVE spectrum of a neural ensemble receiving high synaptic noise (Figure 7 (right, last three rows)), is mainly represented by that of asynchronous spikes. As the synchrony threshold is the same for weak, intermediate, and high synaptic noise, this threshold causes several false-positive synchronous events to be detected when the level of synaptic noise is high. Similar to Figure 6, the TVE spectrum of all spikes for an intermediate level of synaptic noise (see Figure 7 (middle, last three rows)) reveals information underlying slow and fast features of the stimulus. Although the TVE spectrum might not be informative of the decodable information underlying the stimulus when the background synaptic noise level is not biologically realistic (either low or high), the TVE spectrum of all spikes clearly represents that of synchronous and asynchronous spikes for all different levels of synaptic noise.

### 3.4. Relatinship between Mixed Stimulus and Spike Patterns

To identify how synchronous and asynchronous spikes are related to, respectively, the abrupt changes in and the intensity of the stimulus, we develop a decoder model to reconstruct the stimulus from the spikes. A Kalman-filter (KF) decoder model [45,46], a well known state-space approach for neural decoding, is used to reconstruct the stimulus with optimum accuracy underlying linear models [46]. After estimating the parameters of the KF decoder based on mixed stimulus and all spikes, we apply this decoder to synchronous and asynchronous spikes to explore which features of the stimulus are reconstructed. The state-space model of the Kalman-filter decoder can be written as

(14) $$x_{k+1}=Ax_{k}+w$$

(15) $$z_{k}=Hx_{k}+q$$

where $x_{k}$ and $z_{k}\;$ denote the decoded stimulation and the neural firing rate at time index k, respectively. A is the coefficient matrix, w≈N(0,W) represents the uncertainty underlying $x_{k}$, H is a matrix that linearly relates the stimulus to the neural firing, and q≈N(0,Q) is the measurement noise. We estimate the parameter set {A,W,H,Q} from the training data using the following equation.

(16) $$A=\arg min_{A}\;\overset{N-1}{\underset{k=1}{\sum }}{\left|\left|x_{k+1}-Ax_{k}\right|\right|}^{2},H=argmin_{H}\;\overset{N}{\underset{k=1}{\sum }}{\left|\left|z_{k}-Hx_{k}\right|\right|}^{2}$$

**Figure 8:**
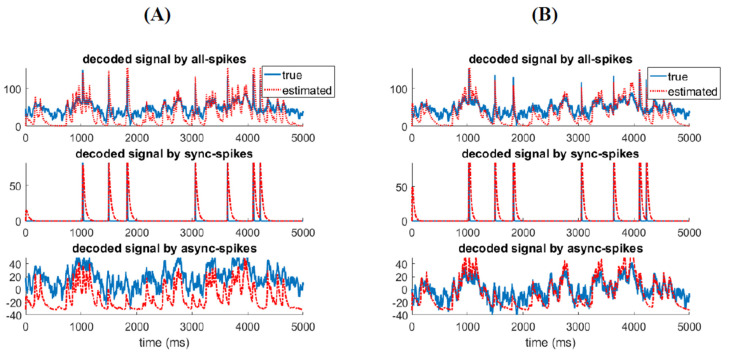
Information decoded by synchronous and asynchronous spikes are associated with different features of the stimulus. (**A**) Kalman-filter decoder model was developed to recunstruct the (mixed) stimulation from all spikes (top). The decoder was then applied to synchronous (middle) and asynchronous spikes (bottom). (**B**) Similar to (**A**) but synchronous (middle) and asynchronous (bottom) spikes were first filtered by a Gausian kernel with optimim time resolutions (δt=0.05 ms for synchronous and δt=10 ms for asynchronous spikes) before applying them to the Kalman-filter decoder model.

By using Equations (14)–(16), we can reconstruct the stimulation from the spiking activity of the ensemble recursively [46]. Figure 8 shows the decoded stimulus from all spikes, synchronous, and asynchronous spikes using the KF-decoder model. Figure 8A shows that we can reconstruct the fast and slow features of the stimulus by applying the decoder model to synchronous and asynchronous spikes, respectively. Furthermore, to enhance the neural decoding, we filtered (using a Gausian kernel) synchronous and asynchronous spikes with optimum time resolutions (δt=0.05 ms and δt=10 ms, respectively) before applying them to the above decoder. Figure 8B shows the reconstructed signals by filtered synchronous and asynchronous spikes. The reconstructed signals are better fitted to the slow and fast features of the stimulus. One can conclude that the information underlying different features of the stimulus is best decoded by different types of spikes, which are integrated with specific time scales.

## 4. Discussion

In this paper, we demonstrated that differentially correlated spikes in a neural ensemble carry different information, which corresponds to different features of the stimulus. By feeding a mixed stimulus consisting of slow and fast features into an ensemble of homogeneous neurons, we created a multiplexed code in which synchronous and asynchronous spikes can be distinguished. It was shown that the probability distribution of these spikes are distinctively separable. Furthermore, we considered spikes as code words and calculated the entropy of these code words for different lengths and time resolutions. A time-varying entropy (TVE) measure was proposed to calculate the entropy of a neural ensemble at each time bin. By applying TVE to the multiplexed code, we showed that information underlying synchronous and asynchronous spikes was maximized for different time resolutions and lengths. Thus, synchronous and asynchronous spikes carried information of different time scales. However, it was observed that the sensitivity of the TVE to the length of codewords was negligible (specifically for high time resolutions). Finally, we developed a Kalman-based decoder to reconstruct the stimulus from the spikes. We showed that slow and fast features of the stimulus could be fully decoded from the asynchronous and synchronous spike, respectively.

As natural stimuli often operate on multiple time scales [47], the TVE calculates the entropy of a homogeneous neural ensemble in different time resolutions, thus providing a time-varying representation of a neural code in different resolution scales. A recent study [47] introduced multiscale relevance (MSR) measure to characterize the temporal structure of the activities of neurons within a heterogeneous population. It was shown that MSR could capture the dynamical variability of the activity of single neurons across different time scales and detect informative neurons as well as neurons that show a high decoding performance [47]. Despite differences in the architecture of the neural ensemble, types of stimuli as well as other factors like the heterogeneity vs. homogeneity of neurons that differentiated between the scope of our study and that of [47], both studies utilized the entropy as an information-theoretic measure and capitalized on the needs of such a measure in multiscale neural analyses.

The advancements of imaging and electrical recording technologies have provided accessibility to neural activities in population levels; thus, the need for multi-dimensional methods is ever increasing in brain-related studies. Time-varying firing rates of a neural ensemble across time and across multiple experimental conditions can be considered as the starting point for the population-level analyses [48]. Kernel smoothing techniques with optimum kernel bandwidth that maximizes the goodness-of-fit of the density estimate to the underlying rate of spikes are tools for estimating the instantaneous firing rate of a neural ensemble [49]. In this regard, one can use the TVE measure as a simple way to identify the most informative time scales underlying the neural code of an ensemble of neurons.

Nevertheless, in population-level analyses, methods that infer the dynamics underlying neural computations are more demanding than those focused on the representation of neuronal activities [48]. Recently, Elsayed and Cunningham [50] proposed a framework to measure the correlation of the neural activity at the population level across times, neurons, and (experimental) conditions. Although this framework was designed for the rate code and thereby cannot be applied to the temporal code, their proposed methodology [50] determines whether neural population activity exhibits a structure above and beyond that of its set of primary features [48]. Unlike [50], the TVE can track the dynamics of a neural code in multiple time scales and one can apply the TVE to both rate and temporal codes simultaneously. Moreover, it is notable that the constraints on the correlation of the neural activity in a neural ensemble across the experimental condition was relaxed in the present study. Taken together, the TVE not only tracks the dynamics of a neural code—in the sense of detecting synchronous and asynchronous states of a neural ensemble—in different time-resolution scales, but also provides decodable information underlying the stimulus features. The TVE can be extended in our future studies to address richer datasets comprising heterogeneous neurons or networks with feed-forward and recurrent connectivity.
